# Elucidating the transcriptomic response of adult-derived mHypoA-2/12 mouse hypothalamic neuron cell line to cannabidiol (CBD) exposure

**DOI:** 10.1007/s13353-025-00970-8

**Published:** 2025-05-08

**Authors:** A. Gurgul, I. Jasielczuk, T. Szmatoła, E. Semik-Gurgul, M. Kucharski, K. Mizera-Szpilka, E. Ocłoń

**Affiliations:** 1 Faculty of Veterinary Medicine, University of Agriculture in Kraków, Redzina 1 C, 30-248, Krakow, Poland; 2 Department of Animal Molecular Biology, National Research Institute of Animal Production, Krakowska 1, 32-083 Balice, Poland; 3 Department of Animal Physiology and Endocrinology, University of Agriculture in Kraków, Mickiewicza 24/28, 30‑059 Krakow, Poland

**Keywords:** CBD, Hypothalamic neurons, Apoptosis, Extracellular matrix, RNA-seq

## Abstract

Cannabidiol (CBD) is a compound found in *Cannabis sativa* that is known for its neuroprotective, anti-inflammatory, analgesic, and anxiolytic properties. These properties make it a promising treatment for various neurological conditions. This study aimed to examine the effects of CBD on hypothalamic neurons at the transcriptome level using the adult-derived mHypoA-2/12 mouse cell line. The cells were exposed to four different CBD concentrations (ranging from 0.325 to 3 µM) for 6 and 24 h. Apart from the transcriptome analysis, apoptosis (caspase 3/7 activity) and viability (MTT) assays were performed. The obtained results showed that CBD enhanced cell viability, especially after 24 h of treatment and at lower or intermediate concentrations, and reduced apoptosis, with significant effects at the highest concentration. CBD caused moderate transcriptome profile changes (13 to 69 genes per treatment), with more genes affected at higher concentrations and shorter exposure times, indicating a stronger initial cellular response. Further analysis revealed that CBD affects several biological processes, including: intrinsic apoptosis suppression via p53 modulation, impacting genes like *Bbc3*, *Mdm2*, *Cdkn1a*, and *Smad3*. Additionally, CBD influenced genes involved in extracellular matrix organization, including metalloproteinases (*Mmp-3*, *Mmp-13*) and their inhibitors (*Timp1*), as well as collagen components (*Col11a1*) and mitochondrial respiratory chain complexes (*mt-Nd5*, *mt-Nd4*). Genes related to serotonin and dopamine biosynthesis, as well as *Aldh2*, were also impacted, linking CBD’s effects in hypothalamic neurons to potential benefits in managing alcohol use disorders. These findings suggest the hypothalamus is a significant target for CBD, warranting further investigation.

**Supplementary Information:**

The online version contains supplementary material available at 10.1007/s13353-025-00970-8.

## Introduction

Cannabidiol (CBD) is one of the major phytocannabinoids found in hemp (*Cannabis sativa*), alongside the more well-known tetrahydrocannabinol (THC) (Meissner and Cascella 2022). Unlike THC, CBD does not have psychotomimetic effects, making it an attractive subject of scientific research and medical interest (Sainz-Cort et al. 2021). CBD has a broad spectrum of potential therapeutic benefits, especially concerning the nervous system (Singh et al. 2023). CBD has neuroprotective potential and anti-inflammatory, analgesic, and anxiolytic properties (Patricio et al. 2020). It is being investigated for use in the treatment of various neurological conditions such as epilepsy, multiple sclerosis, Parkinson’s disease, anxiety, depression, and sleep disorders (de Fátima dos Santos Sampaio et al. 2024). Notably, CBD has gained recognition as an effective treatment for some forms of refractory epilepsy, confirmed by numerous clinical trials and accepted by regulatory agencies such as the US Food and Drug Administration (FDA) (Abu-Sawwa and Stehling 2020) which approved the CBD-based medication Epidiolex for treatment of specific types of epilepsy. CBD actions in the brain are largely complex and not fully understood. Major targeted brain structures include the hippocampus, prefrontal cortex, mesolimbic system, amygdala, caudate, substantia nigra, and hypothalamus (Crippa et al. 2004, 2010; Castillo et al. 2010; Batalla et al. 2020; Lawn et al. 2020).

CBD can potentially act on hypothalamic cells and functions through various molecular mechanisms involving several receptors present in hypothalamic neurons. It has been shown that CBD affects type 1 endocannabinoid (CB1) receptors that are widely distributed throughout the brain, including the hypothalamic suprachiasmatic nucleus (SCN), arcuate nucleus, and paraventricular nucleus (PVN) (Cruz-Martínez et al. 2018). CBD has a low affinity to CB1 and acts as its negative allosteric modulator (Tham et al. 2019). CBD may also modulate CB1 activity indirectly, for example, by affecting the levels of endocannabinoids such as anandamide (Scherma et al. 2019). By inhibiting FAAH (fatty acid amide hydrolase) enzyme, CBD leads to an increase in the level of anandamide that acts on CB1 and CB2 receptors (Leweke et al. 2012). CBD can also serve as an agonist of the 5-HT1 A receptor, which is involved in regulating mood, providing antidepressant, anti-anxiety, and antipsychotic effects (Resstel et al. 2009). Additionally, CBD activates TRPV1 receptors, which are involved in pain processes, thermoregulation, and modulation of neurotransmission (Anand et al. 2020). Other CBD actions in the hypothalamus can be mediated through the activation of PPARγ receptors (Khosropoor et al. 2023). Available data suggests that activation of these receptors in the hypothalamus can affect energy metabolism and appetite regulation (Li et al. 2018). Interestingly, CBD may also act on adenosine receptors, which play a role in regulating sleep (Huang et al. 2014), inflammation (Blackburn et al. 2009), and neuroprotection (Sagredo et al. 2007; Liou et al. 2008; Gonca and Darıcı 2015; Zhang et al. 2020). Finally, CBD may bind with high affinity to the GPR55 receptor (Lauckner et al. 2008; Patricio et al. 2022) and act as an antagonistic ligand, which may affect pain perception (Armin et al. 2021) and inflammation (Ono et al. 2023).

Each of these CBD actions may contribute to the broad spectrum of biological effects in the hypothalamus, highlighting the complexity and multifaceted nature of this compound’s effects on the brain. Specifically, some recent research showed that CBD impacts the synthesis and release of neurotransmitters in the rat Hypo-E22 cells and isolated hypothalamus, including dopamine, norepinephrine, and serotonin (di Giacomo et al. 2020). In the rat ventromedial hypothalamus, CBD has been shown to cause panicolytic-like effects and reduce fear-induced responses, which may be important for managing anxiety-related behaviors (Khan et al. 2020). Furthermore, CBD influences the hypothalamic–pituitary–adrenal (HPA) axis by enhancing its responsivity and normalizing anxiety-like behaviors, particularly in females, suggesting sex-specific effects on stress responses (Jenkins et al. 2025).

Given the fundamental role of gene expression in the hypothalamus’s regulatory functions, this study undertook the analysis of changes arising in hypothalamic cells’ transcriptome following various CBD treatments. For this purpose, we used a hypothalamic cellular model, which allowed us to simplify the complexity of hypothalamic structures and complex interactions with other brain structures, but also abolished the need for animal sacrifice at this stage of experiments. Various hypothalamic cell models, derived from both embryonic (HypoE) and adult primary (HypoA) cultures, are currently available (Dalvi et al. 2011, 2021). These cell lines, immortalized by using a retroviral vector encoding the temperature-sensitive SV40 large T antigen, maintain fundamental characteristics of hypothalamic neurons, including neuronal morphology and expression of hypothalamus-specific markers. To investigate the influence of CBD on transcriptomic profiles of hypothalamic neurons, we employ adult-derived mHypoA-2/12 mouse cell lines. These cell lines have been demonstrated to be valuable models expressing key feeding-related neuropeptides, including NPY and AgRP, along with receptors such as NPY Y1R, NPY Y5R, melanocortin 3 receptor (MC3R), insulin receptor (IR), leptin receptor (Lep-R), ghrelin receptor (GHSR), and glucagon-like peptide receptor type 1 and 2 (GLP-1R, GLP-2R) (Dalvi et al. 2011, 2021).

In the course of the study, the neural cells were treated with different CBD doses and vehicle as a control for 6 and 24 h (h). The experiment setup allowed us to evaluate both dosage and time-dependent effect of CBD on hypothalamic cells, particularly on their viability, apoptosis and transcriptome profile, including specific genes expression. The transcriptome-wide method we employed enabled the formulation of a simplified, general hypothesis that CBD impacts the cells’ transcriptome. Our detailed analysis focused on identifying the altered pathways and associated biological processes. The results showed coordinated gene expression changes across different CBD treatments, revealing some of the mechanisms of CBD action in hypothalamic neural cells.

## Material and methods

### Cell culture and treatments

The adult mouse hypothalamic cell lines mHypoA-2/12 (Clu177) were purchased from Cedarlane (Canada) and cultured in 6 well plates (Thermo Fisher Scientific, USA) with Dulbecco’s Modified Eagle’s Medium with GlutaMAX (DMEM, 4500 mg/L, Gibco—Thermo Fisher Scientific, USA) supplemented with 10% fetal bovine serum (FBS, Gibco, Thermo Fisher Scientific, USA) and 1% Gibco™ Antibiotic–Antimycotic (10,000 units/mL of penicillin, 10,000 μg/mL of streptomycin, and 25 μg/mL of Gibco Amphotericin B, Thermo Fisher Scientific, USA) maintained at 37 °C and 5% CO^2^ until 75% confluency. The following day, the cells were cultured in DMEM with GlutaMAX, supplemented with CBD (0.325, 0.75, 1.5, and 3 µM) (MERCK, USA) either for 6 h (n = 3 for each concentration) or for 24 h (n = 3 for each concentration). CBD was suspended in DMSO and diluted to a final concentration in DMEM with GlutaMAX (final DMSO concentration of 0.1% in assay media). The vehicle control was 0.1% DMSO in the DMEM with GlutaMAX.

### Cellular tests

#### Cell viability assay

Cell viability assays were performed on separate cell cultures treated at the same time as ones for RNA-Seq using the RealTime-Glo™ MT Cell Viability Assay according to the manufacturer’s protocol (Promega, USA). Cells were seeded at a density of 4000 cells/well into white-walled 96-well opaque assay plates. After the plated cells were allowed to attach overnight, they were treated with CBD concentrations ranging from 0.325 to 3 µM. The MT Cell Viability Substrate and NanoLuc Enzyme were equilibrated to 37 °C, 2 × RealTime-Glo reagent was prepared, and an equal volume was added to each well. For time zero measurements, cells were incubated with reagent for 20 min at 37 °C, and luminescence intensity was determined on a TECAN Infinite M200 PRO microplate reader. Luminescence was measured at 6 and 24 h after the addition of CBD.

#### Caspase activity

Caspase 3/7 activity was determined according to the manufacturer’s protocol. Briefly, mHypoA-2/12 cells (15 × 10^3^ cells per well) were grown in a 96-well white plate and exposed to CBD for 6 h or 24 h. Cells with the addition of 5 μM staurosporine (0.1% final DMSO; Merck, USA) for 6 h were used as a positive control. For the assay, Caspase-Glo 3/7 reagent was added to all the wells in a 1:1 ratio and after shaking at room temperature for 30 min, the lysates were analyzed with the luminometer (Infinite M200 PRO, TECAN, Switzerland).

### Transcriptome analysis

After the treatment time elapsed, cells were trypsinized, transferred to centrifuge tubes, and centrifuged at 500 RCF for 5 min to form pellets. Following removal of the supernatant, the cell pellets were snap-frozen at − 80 °C and stored until RNA isolation. Total RNA was isolated using the standard TRI Reagent™ Solution (ThermoFisher Scientific, USA) procedure. The quality of the RNA isolates was evaluated using the TapeStation 4150 System (Agilent, USA), and RNA quantification was performed using the Qubit RNA BR assay (ThermoFisher Scientific, USA).

Library preparation was carried out with 50 ng of total RNA using the QuantSeq 3′ mRNA-Seq Library Prep Kit FWD (Lexogen, Austria). This kit generates one fragment per transcript at the 3′ end, reducing the number of raw reads generated per sample to approximately 3 million (M). The quality of the indexed libraries was checked using the TapeStation 4150 System (Agilent, USA) and quantified using the Qubit dsDNA BR kit (ThermoFisher Scientific, USA). Finally, the library pools were sequenced commercially by the OMRF Clinical Genomics Center (CGC) in a single-end 150 bp run on a NovaSeq 6000 System (Illumina, USA), yielding at least 6–7 M reads per sample. These raw sequences and read counts were deposited in the Gene Expression Omnibus (GEO) and Sequence Read Archive (SRA) databases of the National Center for Biotechnology Information (NCBI) under accession number GSE270378.

### Bioinformatic analysis

The raw sequencing reads received were quality-checked using FastQC (v0.11.9) software. Filtering and trimming were done using Flexbar software (3.5.0) (Dodt et al. 2012). During filtering, low-quality read ends, adapter sequences, and reads that were too short after trimming were removed. High-quality cleaned reads were mapped to the Mouse GRC39 genome assembly using the STAR aligner software (2.7.5c) (Dobin et al. 2013). Mapped reads were counted using MM109 (Ensembl) annotation and Htseq-count (1.99.2) (Anders et al. 2015) software. Normalization of red counts and differential expression (DE) analysis were performed using DESeq2 (v3.16) (Love et al. 2014) software. An FDR < 0.1 was required to consider a gene as differentially expressed. The DE genes were analyzed for their functional implications by enrichment analysis in a specific GO BP (gene ontology biological processes), KEEG (Kyoto Encyclopedia of Genes and Genomes), and GO CC (cellular components) categories using iDEP2.0 server (Ge et al. 2018). Additional functional enrichment analysis was performed with WebGestalt (WEB-based GEne SeT AnaLysis Toolkit) (Liao et al. 2019). Venn diagrams were prepared using online tools (https://bioinformatics.psb.ugent.be/webtools/Venn/) (Heberle et al. 2015).

### qPCR validation of 3′mRNA-Seq

Due to the insufficient amount or total RNA from initial treatments and purification, qPCR validation of 3′mRNA-Seq results was conducted based on replicated experiments on the same cell line. The cells were treated as in the main experiment with all CBD doses and vehicle as control. Only 24-h treatment was replicated. For validation, four genes that were differently expressed in at least three comparisons (treatments) in a major experiment (in 24-h treatment) were selected. The genes included *Mmp13*, *Naca*, *Ifitm2*, and *Gm8960*. The *Hprt* gene was used as an endogenous control (Zamani et al. 2020). Primer sequences are provided in Supplementary File 1.

Genes were tested using RT-qPCR to verify the reliability of the analysis. For this purpose, cDNA was synthesized using 500 ng of RNA and the High-Capacity RNA-to-cDNA kit (Thermo Fisher Scientific), following the manufacturer’s protocol. RT-qPCR was performed with the AmpliQ 5 × HOT EvaGreen® qPCR Mix Plus (ROX) kit (Novazym, Poznan, Poland) and primers for mRNA sequences spanning two adjacent exons. Each sample was run in triplicate using Quant Studio 7 Flex (Thermo Fisher Scientific). The relative expression levels of each gene were calculated using the ΔΔCt method. Standardization was performed based on *Hprt* as the internal control.

Relative expression concordance between RNA-Seq and qPCR methods was assessed based on the correlation coefficient analysis on mean expression values within groups. Calculations were performed using JASP 0.11.1 software. The Spearman correlation coefficient was selected for this analysis, following data distribution evaluation using the Shapiro–Wilk test.

## Results

### Effect of CBD exposure on cells viability and apoptosis

**Fig. 1:**
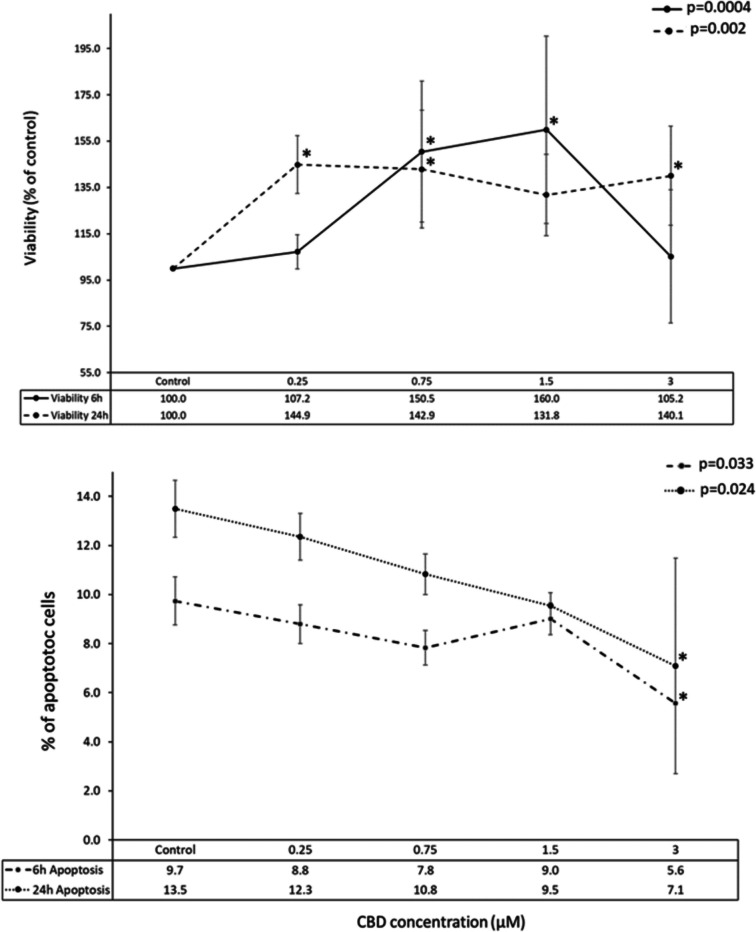
Results of caspase 3/7 and MMT assay for cells treated with various concentrations of CBD. *P*-values of the Kruskal–Wallis ANOVA test are provided. *Statistically significant difference (adjp < 0.05) after correction for multiple testing for comparison of a specific CBD concentration against vehicle control

To assess the impact of CBD on mHypoA-2/12 cell viability, the cells were treated with increasing concentrations of CBD (0.25, 0.75, 1.5, and 3 µM) for 6 or 24 h. Notably, within 24 h of exposure, CBD significantly enhanced cell survival across all concentrations (Fig. 1; *p* < 0.002 vs. vehicle control). As illustrated in Fig. 1, there is a notable increase in cell viability after 6 h of CBD treatment, particularly with concentrations of 0.75 and 1.5 µM (Fig. 1; *p* < 0.0004).

To investigate whether CBD-induced cell death occurred via apoptosis, we measured caspase 3/7 activity. Interestingly, at the highest CBD concentration (3 µM), caspase 3/7 activity significantly decreased after both 6 and 24 h (Fig. 1; *p* < 0.033 and *p* < 0.024, respectively). However, no significant changes in caspase 3/7 activity were observed in the remaining experimental conditions.

### Sequencing statistics and general genes expression profile differentiation

In total, for 3′mRNA-Seq experiment, more than 210 M sequencing reads were generated for 30 samples—around 7 M per sample on average. Of the raw reads, on average 98.9% passed initial filtering and 54% were uniquely mapped against the newest available reference genome sequence. More than 75% of the mapped reads were located within annotation features (genes) (Supplementary File 2). Gene-related read counts were further used for differential expression analysis between various experimental conditions and transcriptome profile differentiation analysis.

**Fig. 2:**
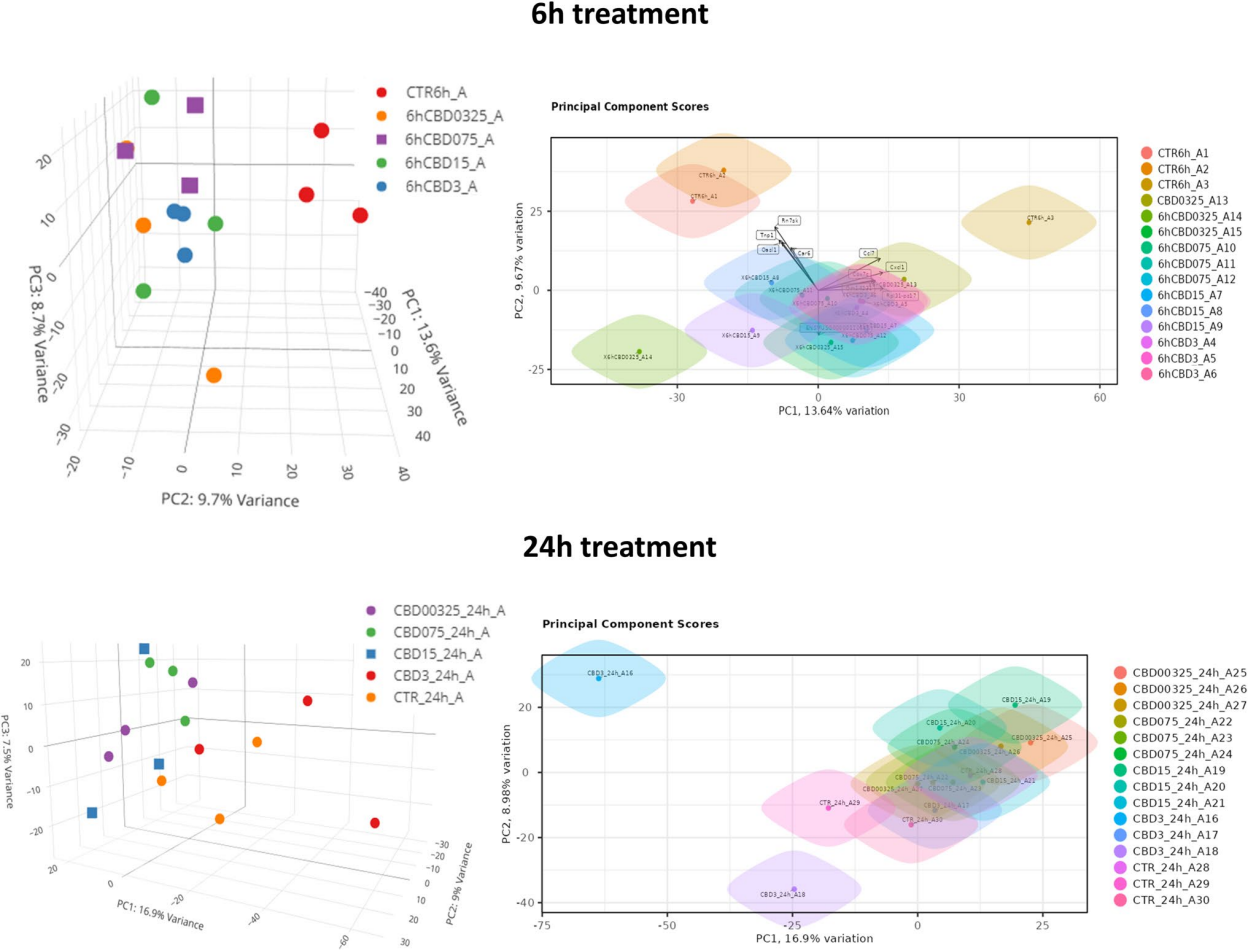
Expression profile differentiation using principal components analysis (PCA) method among cells treated with different CBD concentrations and after different times of CBD treatment

In 6-h treatment, CBD supplementation at various concentrations induced only a minor change in neural cells transcriptomes, but genially, expression profile of treated cells differed form control cells (vehicle). This was visible in principal components analysis (PCA) in which control cells clustered separately from a mixed group of cells treated with different concentrations of CBD. At 24-h treatment, the separation of control cells from treatments was not so clearly visible, and they co-localized in the PCA graph with 3 µM CBD-treated cells (Fig. 2).

**Fig. 3:**
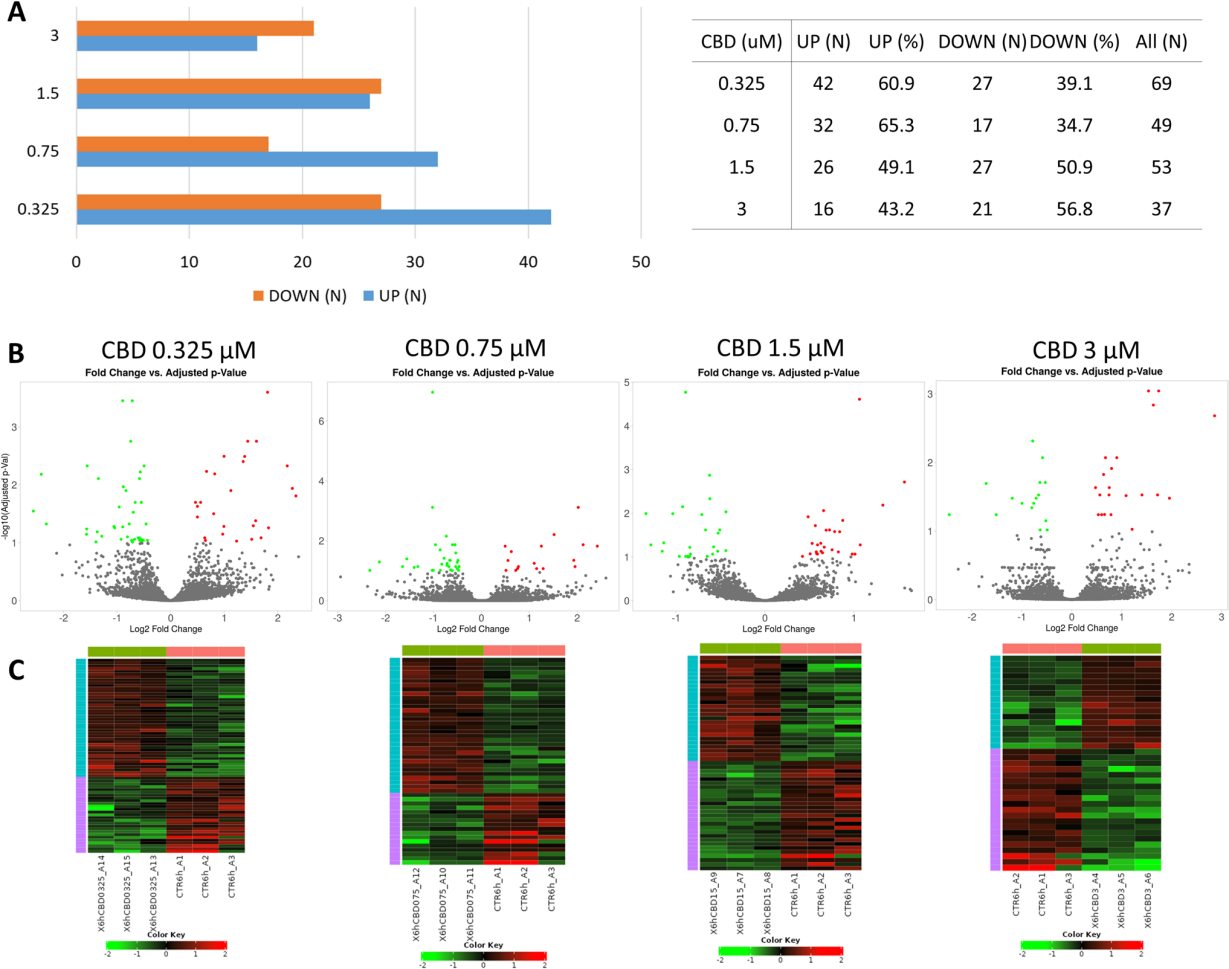
Results of differential expression analysis among cells treated with different concentrations of CBD for 6 h. **A** Number of differentially expressed genes and their regulation; **B** volcano plot for all analyzed genes (significantly altered genes (FDR < 0.1) are marked in red/green); **C** heatmaps for differentially expressed genes

In 6-h treatment, the highest amount of genes was altered by the lowest applied CBD concentration (0.325 µM; *n* = 369) and in contrast, the lowest number of genes was altered by the highest CBD dose (3 µM; *n* = 37) (Supplementary File 3). At lower CBD concentrations, the genes were mainly downregulated (60.9 and 65.3% for 0.325 and 0.7 µM CBD, respectively), while the 3 μM CBD dose was associated with predominant genes upregulation (56.8%) (Fig. 3).

**Fig. 4:**
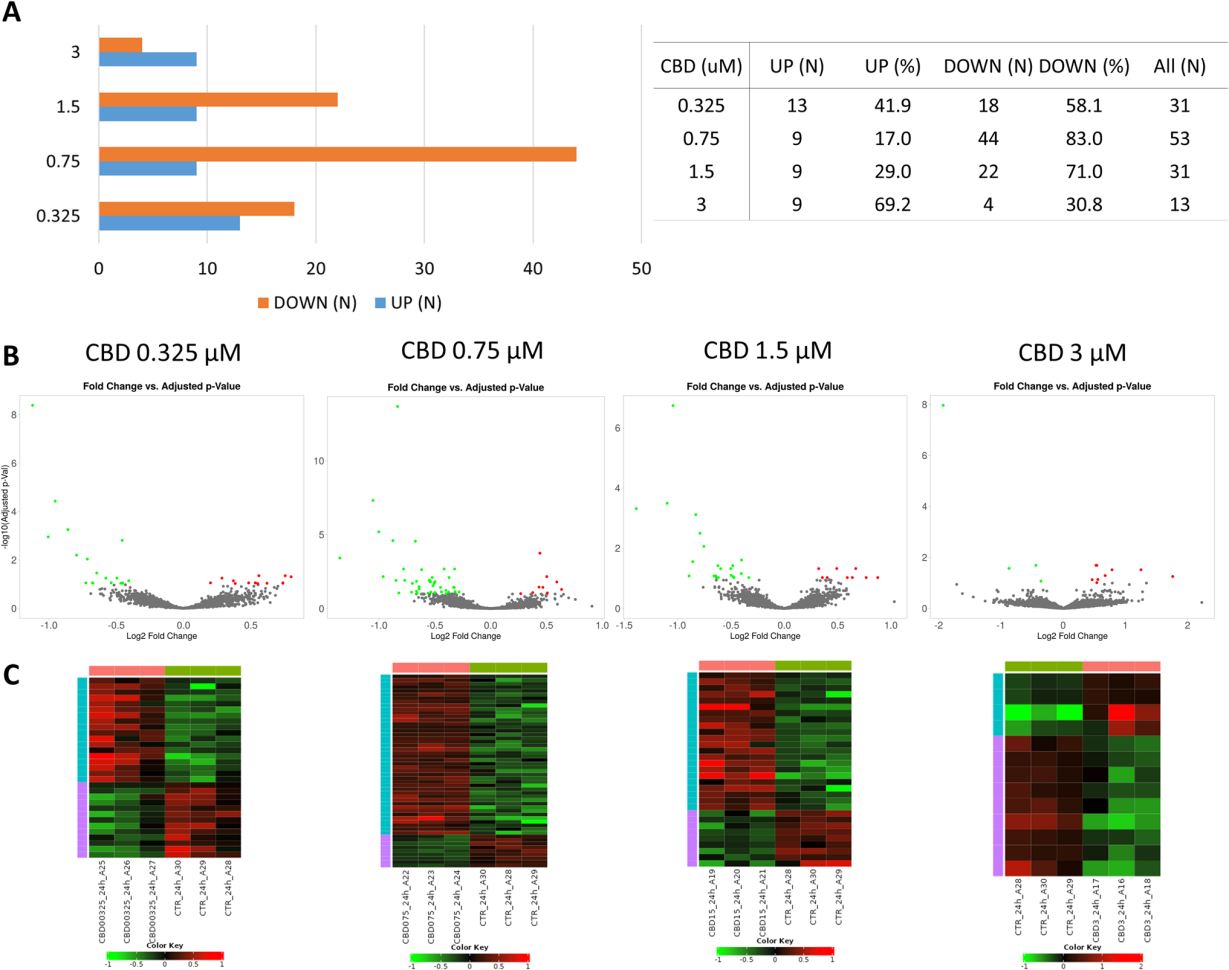
Results of differential expression analysis among cells treated with different concentrations of CBD for 24 h. **A** Number of differentially expressed genes and their regulation; **B** volcano plot for all analyzed genes (significantly altered genes (FDR < 0.1) are marked in red/green); **C** heatmaps for differentially expressed genes

Following 24 h of treatment, again the highest amount of genes was altered by lower CBD concentrations (0.0325 and 0.075 µM; *n* = 331 and 53, respectively) (Supplementary File 3). The lowest amount of altered genes was observed for cells treated with 3 µM of CBD (*n* = 313). In the most treatments, (except 3 µM CBD), CBD caused mainly downregulation of genes expression (Fig. 4).

**Fig. 5:**
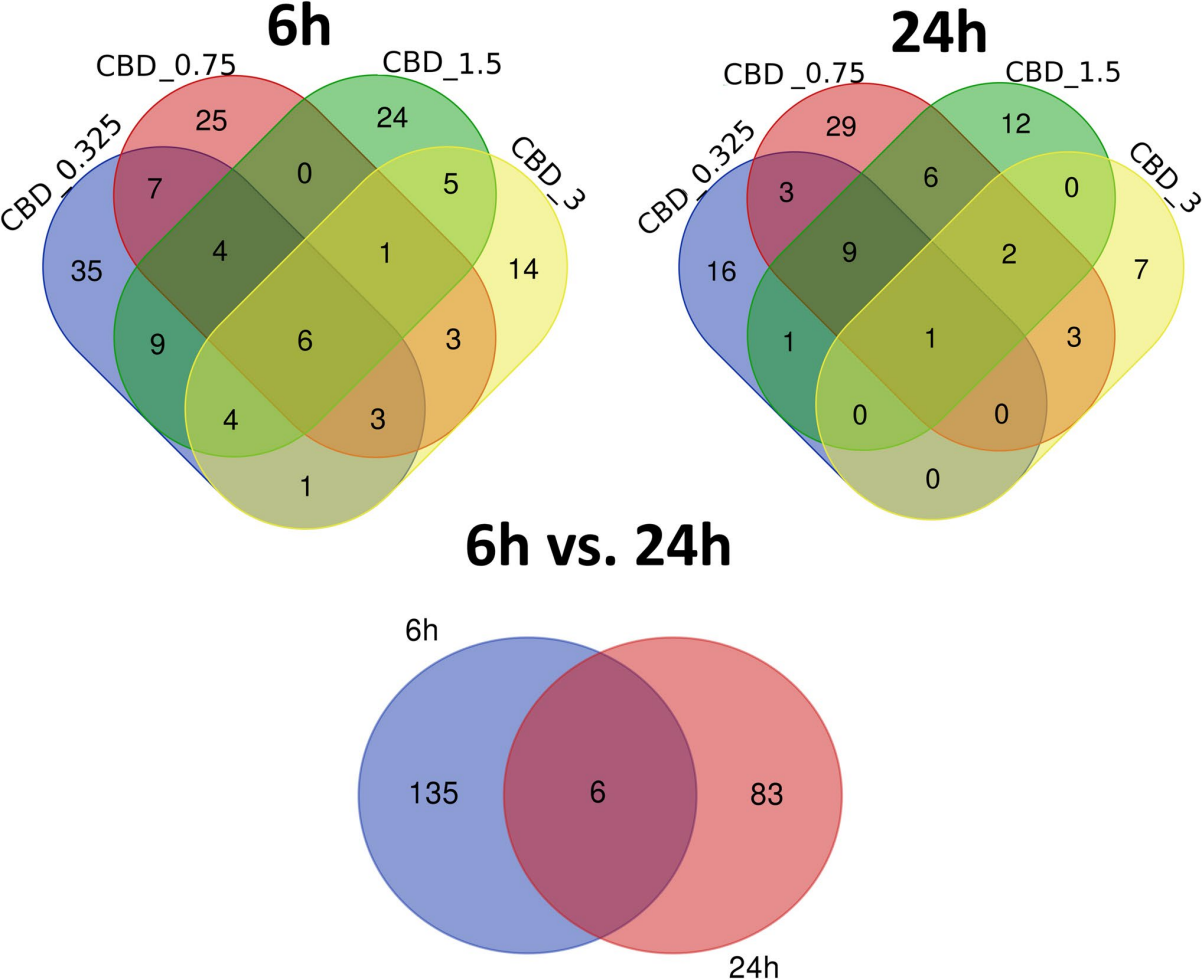
Venn diagrams for genes altered by different CBD treatments (concentrations) and treatment times

Of the 141 different genes whose expression was altered by CBD in 6-h treatment, six were affected by all applied CBD concentrations, and other 11 by at least three different CBD doses. The cells treated with 3 μM CBD solution had the most distinct profile of genes expression changes showed by the lowest amount of genes that were commonly altered by other treatments (Fig. 5). After 24-h incubation with CBD, of the 89 altered genes, only one was common for all treatments, however nine others were common for three lower CBD doses. All those nine genes were downregulated (Fig. 5). What is more, six common genes were altered in CBD-treated cells at 6 and 24 h (Supplementary File 4).

#### Functional annotation of genes altered by CBD in neural cells

**Fig. 6:**
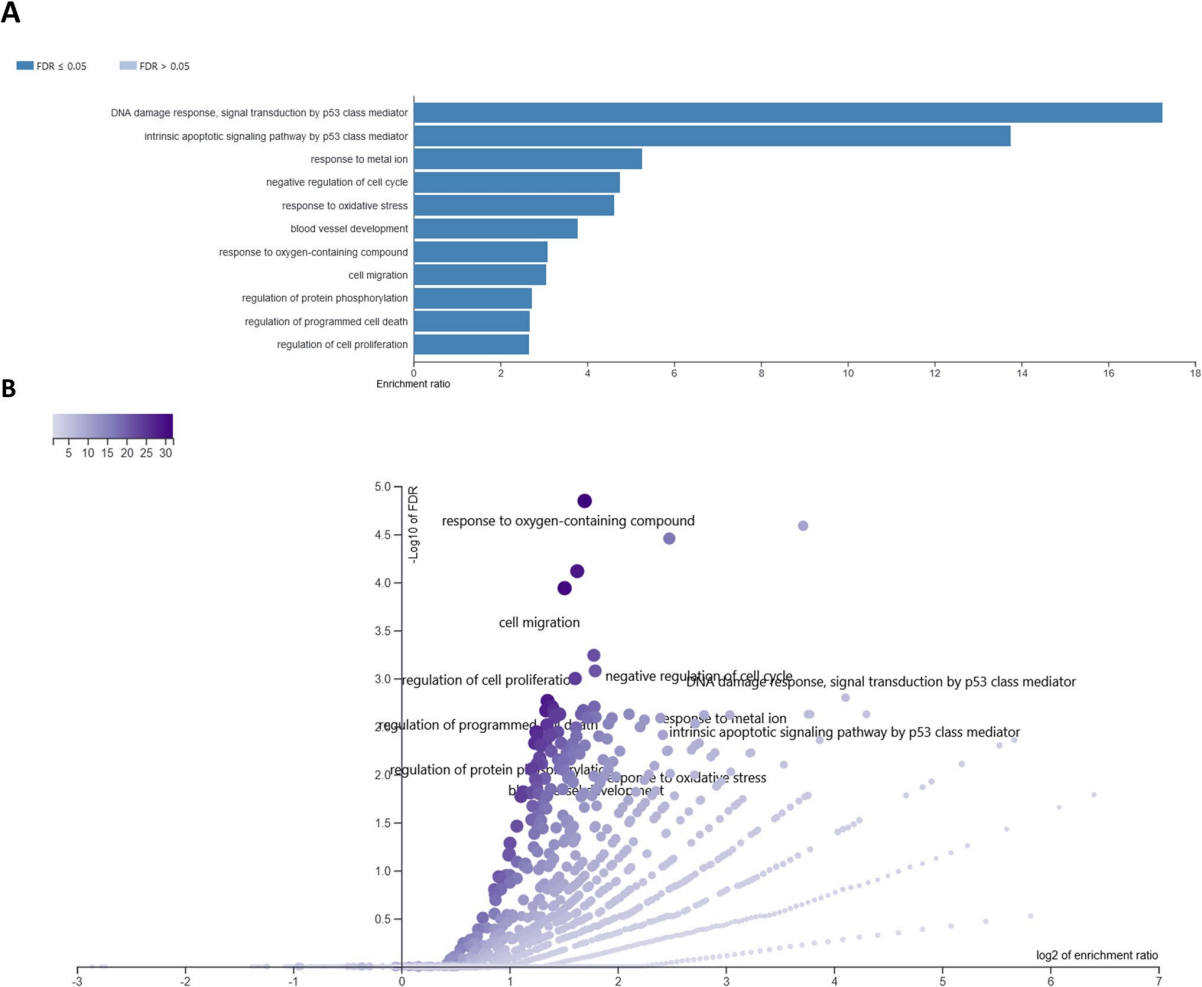
Result of overrepresentation test in GO biological processes for all genes affected by CBD in 6-h treatment

After 6-h treatment of neural cells with different concentrations of CBD, in total 141 different genes were affected, from 37 (3 μM CBD) to 69 (0.325 μM) per treatment. The overrepresentation tests for those genes in GO BP and KEEG categories are presented in Supplementary File 5. Among top 50 overrepresented (FDR < 0.1) BP and KEEG pathways for lower CBD concentrations, only processes associated with upregulated genes have been found, however, in case of 3 μM concentration, both up- and downregulated genes were enriched. A comparative analysis of GO BP among various CBD treatments showed, that processes such as cell population proliferation, immune system process, cellular response to stress, cellular response to decreased oxygen levels, cellular response to hypoxia, DNA damage response signal transduction by p53 class mediator, positive regulation of programmed cell death, apoptotic process, and positive regulation of metabolic process were overrepresented by genes altered by at least three different CBD treatments. A similar analysis performed for KEEG pathways showed that pathways such as pathways in cancer, P53 signaling, and osteoclast differentiation were found to be overrepresented for at least three different CBD treatments (Supplementary File 5; Table 1). Comparative analysis of the enriched (FDR < 0.1) cellular components (Supplementary File 6) showed that categories related to, e.g., PCNA-p21 complex, PUMA-BCL-xl complex, nucleolus, extracellular region, extracellular matrix, collagen-containing extracellular matrix, external encapsulating structure, and extracellular space were associated with genes altered by more than one CBD treatment (Supplementary File 6). Combined analysis of all 141 genes altered by CBD in 6-h treatment revealed that (following redundancy removal by affinity propagation) the enriched BP (FDR < 0.1) included among others: response to oxygen-containing compound, cell migration, regulation of cell proliferation, intrinsic apoptotic signaling pathway by p53 class mediator, regulation of programmed cell death, response to metal ion, negative regulation of cell cycle, response to oxidative stress, and others. Detailed information on this analysis can be found in Supplementary File 7 and Fig. 6. In depth analysis of six genes that were altered by all CBD concentrations at 6-h treatment, and other 11 that were common for at least three different CBD doses (Supplementary File 4) showed that the genes were enriched (FDR < 0.1) in BP such as: wound healing involved in inflammatory response, inflammatory response to wounding and cellular response to stress. They also included two genes directly involved in extracellular matrix functioning, namely *Mmp3* and *Timp1* genes.

**Table 1:** Comparative analysis of biological processes and KEEG pathways overrepresented by different CBD treatments (concentrations) at 6-h treatment. Processes overrepresented by at least two treatments are presented along with the engaged genes

6-h treatment		
Common for CBD treatment	Category	Genes
**Biological processes**		
CBD_0.325 CBD_0.75 CBD_1.5 CBD_3	Cell population proliferation	ENSMUSG00000052837, ENSMUSG00000022528, ENSMUSG00000005125, ENSMUSG00000005413, ENSMUSG00000023034, ENSMUSG00000023067, ENSMUSG00000027803, ENSMUSG00000032402, ENSMUSG00000015837, ENSMUSG00000024190, ENSMUSG00000020184, ENSMUSG00000040435, ENSMUSG00000022528, ENSMUSG00000052684, ENSMUSG00000029377, ENSMUSG00000052837, ENSMUSG00000041313, ENSMUSG00000056708, ENSMUSG00000038418, ENSMUSG00000030717, ENSMUSG00000032501
	Immune system process	ENSMUSG00000090272, ENSMUSG00000005125, ENSMUSG00000005413, ENSMUSG00000015837, ENSMUSG00000022528, ENSMUSG00000024190, ENSMUSG00000029561, ENSMUSG00000032402, ENSMUSG00000034459, ENSMUSG00000035692, ENSMUSG00000041827, ENSMUSG00000023067, ENSMUSG00000052837, ENSMUSG00000052684, ENSMUSG00000027111, ENSMUSG00000090272, ENSMUSG00000021250, ENSMUSG00000039883, ENSMUSG00000041313, ENSMUSG00000029377, ENSMUSG00000038418, ENSMUSG00000073489, ENSMUSG00000032501, ENSMUSG00000042406,
	Regulation of cell population proliferation	ENSMUSG00000052837, ENSMUSG00000022528, ENSMUSG00000005125, ENSMUSG00000005413, ENSMUSG00000023034, ENSMUSG00000023067, ENSMUSG00000027803, ENSMUSG00000032402, ENSMUSG00000024190, ENSMUSG00000020184, ENSMUSG00000040435, ENSMUSG00000022528, ENSMUSG00000052684, ENSMUSG00000029377, ENSMUSG00000052837, ENSMUSG00000041313, ENSMUSG00000056708, ENSMUSG00000038418, ENSMUSG00000030717, ENSMUSG00000032501,
	Cellular response to stress	ENSMUSG00000002083, ENSMUSG00000024190, ENSMUSG00000040435, ENSMUSG00000023067, ENSMUSG00000005125, ENSMUSG00000005413, ENSMUSG00000015837, ENSMUSG00000020184, ENSMUSG00000021453, ENSMUSG00000021701, ENSMUSG00000032402, ENSMUSG00000052684, ENSMUSG00000009630, ENSMUSG00000023067, ENSMUSG00000038418, ENSMUSG00000021250, ENSMUSG00000028494, ENSMUSG00000056708, ENSMUSG00000024843, ENSMUSG00000040435, ENSMUSG00000042406, ENSMUSG00000030717, ENSMUSG00000032501, ENSMUSG00000073489,
CBD_0.325 CBD_0.75 CBD_1.5	Cellular response to decreased oxygen levels	ENSMUSG00000002083, ENSMUSG00000005125, ENSMUSG00000005413, ENSMUSG00000020184 ENSMUSG00000052684, ENSMUSG00000038418, ENSMUSG00000021250,
	Cellular response to hypoxia	ENSMUSG00000002083, ENSMUSG00000005125, ENSMUSG00000005413, ENSMUSG00000020184, ENSMUSG00000052684, ENSMUSG00000038418, ENSMUSG00000021250
	DNA damage response signal transduction by p53 class mediator	ENSMUSG00000020184, ENSMUSG00000023067, ENSMUSG00000023067, ENSMUSG00000005125
CBD_0.325 CBD_0.75 CBD_3	Positive regulation of programmed cell death	ENSMUSG00000023034, ENSMUSG00000002083, ENSMUSG00000021453, ENSMUSG00000005413, ENSMUSG00000023067, ENSMUSG00000024190, ENSMUSG00000040435, ENSMUSG00000052684, ENSMUSG00000030717, ENSMUSG00000042406,
	Negative regulation of cell population proliferation	ENSMUSG00000022528, ENSMUSG00000005413, ENSMUSG00000023067, ENSMUSG00000032402, ENSMUSG00000005125, ENSMUSG00000024190, ENSMUSG00000040435, ENSMUSG00000052684, ENSMUSG00000030717, ENSMUSG00000032501,
	Response to stress	ENSMUSG00000002083, ENSMUSG00000005413, ENSMUSG00000024190, ENSMUSG00000029561, ENSMUSG00000034459, ENSMUSG00000040435, ENSMUSG00000041827, ENSMUSG00000090272, ENSMUSG00000023067, ENSMUSG00000005125, ENSMUSG00000015837, ENSMUSG00000020184, ENSMUSG00000021453, ENSMUSG00000032402, ENSMUSG00000035692, ENSMUSG00000021701, ENSMUSG00000052684, ENSMUSG00000009630, ENSMUSG00000023067, ENSMUSG00000038418, ENSMUSG00000021250, ENSMUSG00000028494, ENSMUSG00000056708, ENSMUSG00000024843, ENSMUSG00000005413, ENSMUSG00000073489, ENSMUSG00000042406, ENSMUSG00000030717, ENSMUSG00000032501
	Apoptotic process	ENSMUSG00000002083, ENSMUSG00000020184, ENSMUSG00000023034, ENSMUSG00000005413, ENSMUSG00000015837, ENSMUSG00000021453, ENSMUSG00000032402, ENSMUSG00000040435, ENSMUSG00000021701, ENSMUSG00000023067, ENSMUSG00000024190, ENSMUSG00000052684, ENSMUSG00000009630, ENSMUSG00000042406, ENSMUSG00000003541, ENSMUSG00000030717, ENSMUSG00000073489
	Cell death	ENSMUSG00000002083, ENSMUSG00000020184, ENSMUSG00000023034, ENSMUSG00000005413, ENSMUSG00000015837, ENSMUSG00000021453, ENSMUSG00000032402, ENSMUSG00000040435, ENSMUSG00000021701, ENSMUSG00000023067, ENSMUSG00000024190, ENSMUSG00000052684, ENSMUSG00000009630, ENSMUSG00000042406, ENSMUSG00000003541, ENSMUSG00000030717, ENSMUSG00000073489
	Programmed cell death	ENSMUSG00000002083, ENSMUSG00000020184, ENSMUSG00000023034, ENSMUSG00000005413, ENSMUSG00000015837, ENSMUSG00000021453, ENSMUSG00000032402, ENSMUSG00000040435, ENSMUSG00000021701, ENSMUSG00000023067, ENSMUSG00000024190, ENSMUSG00000052684, ENSMUSG00000009630, ENSMUSG00000042406, ENSMUSG00000003541, ENSMUSG00000030717, ENSMUSG00000073489
	Signal transduction by p53 class mediator	ENSMUSG00000023067, ENSMUSG00000005125, ENSMUSG00000020184, ENSMUSG00000002083 ENSMUSG00000030717, ENSMUSG00000073489
CBD_0.325 CBD_1.5 CBD_3	Positive regulation of metabolic process	ENSMUSG00000002083, ENSMUSG00000027803, ENSMUSG00000040435, ENSMUSG00000090272, ENSMUSG00000022528, ENSMUSG00000023034, ENSMUSG00000032402, ENSMUSG00000000078, ENSMUSG00000015837, ENSMUSG00000020184, ENSMUSG00000021701, ENSMUSG00000023067, ENSMUSG00000035692, ENSMUSG00000052837, ENSMUSG00000005413, ENSMUSG00000029377, ENSMUSG00000038418, ENSMUSG00000021250, ENSMUSG00000027111, ENSMUSG00000056708, ENSMUSG00000001627, ENSMUSG00000032501, ENSMUSG00000038508, ENSMUSG00000073489, ENSMUSG00000042406, ENSMUSG00000030717,
**KEEG**		
CBD_0.325 CBD_0.75 CBD_1.5	Colorectal cancer	ENSMUSG00000023067, ENSMUSG00000002083, ENSMUSG00000032402, ENSMUSG00000021453, ENSMUSG00000052684, ENSMUSG00000029377, ENSMUSG00000021250
	Human papillomavirus infection	ENSMUSG00000023067, ENSMUSG00000022528, ENSMUSG00000020184, ENSMUSG00000041827, ENSMUSG00000029561, ENSMUSG00000009630, ENSMUSG00000027111, ENSMUSG00000026478,
	Mitophagy-animal	ENSMUSG00000015837, ENSMUSG00000008348, ENSMUSG00000052684, ENSMUSG00000015837
	Pathways in cancer	ENSMUSG00000023067, ENSMUSG00000022528, ENSMUSG00000005413, ENSMUSG00000002083, ENSMUSG00000032402, ENSMUSG00000020184, ENSMUSG00000021453, ENSMUSG00000052684, ENSMUSG00000021250, ENSMUSG00000027111, ENSMUSG00000026478
	P53 signaling pathway	ENSMUSG00000023067, ENSMUSG00000002083, ENSMUSG00000020184, ENSMUSG00000021453,
	Platinum drug resistance	ENSMUSG00000023067, ENSMUSG00000002083, ENSMUSG00000020184
	Epstein-Barr virus infection	ENSMUSG00000023067, ENSMUSG00000022528, ENSMUSG00000020184, ENSMUSG00000021453, ENSMUSG00000052684
	Breast cancer	ENSMUSG00000023067, ENSMUSG00000022528, ENSMUSG00000021453 ENSMUSG00000052684, ENSMUSG00000021250,
	Osteoclast differentiation	ENSMUSG00000052837, ENSMUSG00000015837, ENSMUSG00000003545, ENSMUSG00000052684, ENSMUSG00000021250, ENSMUSG00000052837,
CBD_0.325 CBD_1.5	Endocrine resistance	ENSMUSG00000023067, ENSMUSG00000020184, ENSMUSG00000021250
	Maturity onset diabetes of the young	ENSMUSG00000022528
	Melanoma	ENSMUSG00000023067, ENSMUSG00000020184, ENSMUSG00000021453
	Prostate cancer	ENSMUSG00000023067, ENSMUSG00000020184,
	Small cell lung cancer	ENSMUSG00000023067, ENSMUSG00000021453, ENSMUSG00000027111, ENSMUSG00000026478,
	Cellular senescence	ENSMUSG00000023067, ENSMUSG00000032402, ENSMUSG00000020184, ENSMUSG00000015837, ENSMUSG00000021453
	Bladder cancer	ENSMUSG00000023067, ENSMUSG00000020184,
	HIF-1 signaling pathway	ENSMUSG00000023067, ENSMUSG00000005413,
	MicroRNAs in cancer	ENSMUSG00000023067, ENSMUSG00000005413, ENSMUSG00000020184, ENSMUSG00000041313, ENSMUSG00000023067
	Glioma	ENSMUSG00000023067, ENSMUSG00000020184, ENSMUSG00000021453
	Chronic myeloid leukemia	ENSMUSG00000023067, ENSMUSG00000032402, ENSMUSG00000020184, ENSMUSG00000021453,
	Fluid shear stress and atherosclerosis	ENSMUSG00000005413, ENSMUSG00000015837, ENSMUSG00000024190, ENSMUSG00000021250, ENSMUSG00000005413
CBD_0.325 CBD_3	Transcriptional misregulation in cancer	ENSMUSG00000023067, ENSMUSG00000020184, ENSMUSG00000021453, ENSMUSG00000055435, ENSMUSG00000043613

**Fig. 7:**
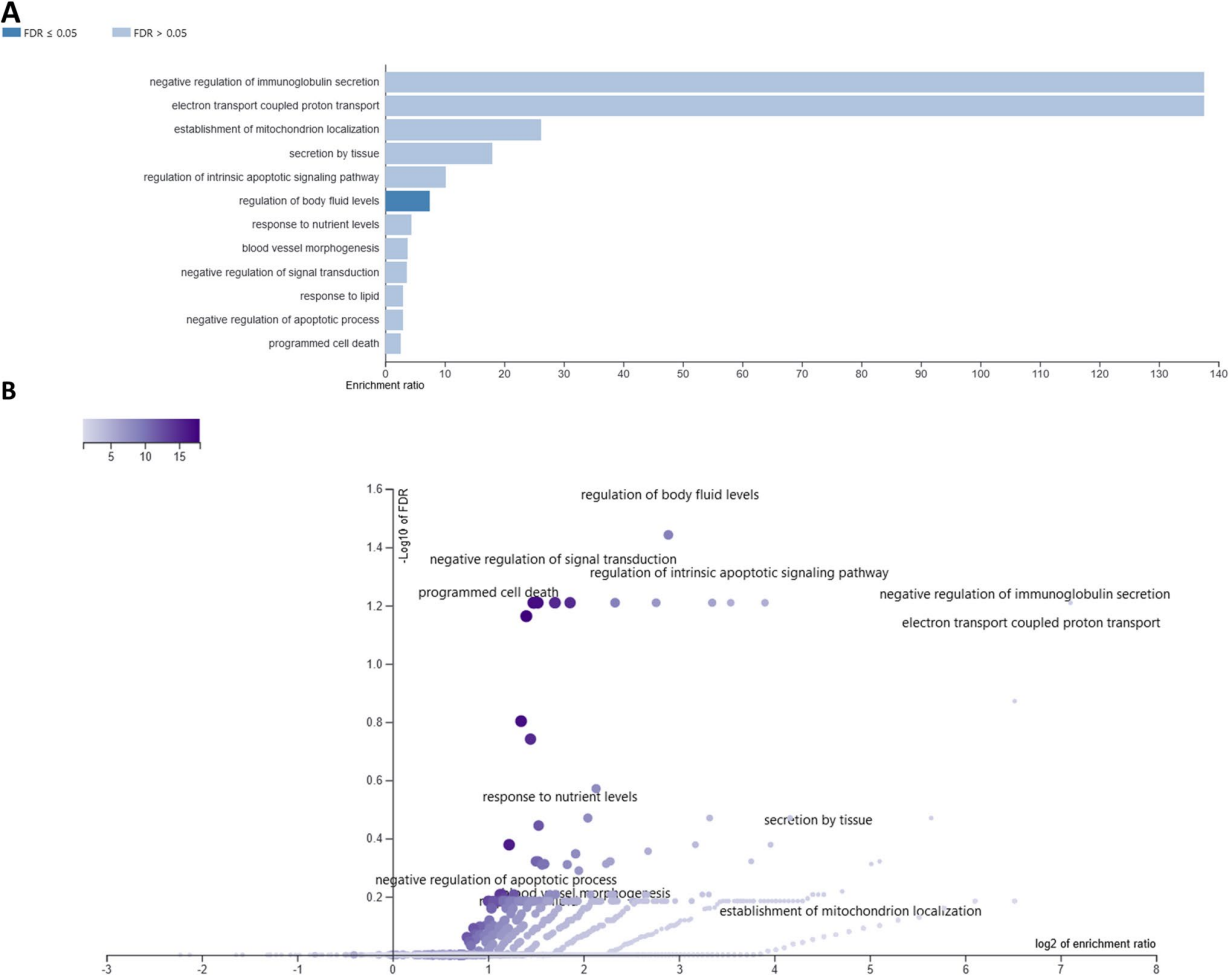
Result of overrepresentation test in GO biological processes for all genes affected by CBD in 24-h treatment

After 24 h CBD treatment, 89 different genes were altered with respect to vehicle (control), from 13 (3 μM) to 53 (0.75 μM) per treatment. Among the top 50 enriched BP (Supplementary File 8) comparative analysis revealed: cell motility and migration, acute-phase response, monocyte chemotaxis, negative regulation of leukocyte migration, and amine transport-related biological processes, as overrepresented by at least two different CBD treatments. Among the common for different treatments overrepresented (FDR < 0.1) KEEG pathways, there were, e.g., Parkinson disease, thermogenesis, RNA degradation, vascular smooth muscle contraction, platelet activation, oxytocin signaling pathway, and calcium signaling pathway (Supplementary File 8; Table 2). Only genes altered by two extreme CBD doses enriched any cellular components (CC) categories. Genes affected by 0.325 μM CBD treatment enriched CC associated with, e.g., mitochondrial respiratory chain complex I, NADH dehydrogenase complex (upregulated genes) and extracellular space, cell–cell junction, intercellular canaliculus (downregulated genes) and others (Supplementary File 6). 3 μM CBD dose, however, affected genes that were enriched in CC such as elongator holoenzyme complex, organellar ribosome, stress fiber and mitochondrial ribosome (Supplementary File 6). Combined analysis of all 89 CBD-affected genes in 24-h treatment revealed that following affinity propagation redundancy removal several processes remained enriched (FDR < 0.1), namely, regulation of body fluid levels, negative regulation of signal transduction, regulation of intrinsic apoptotic signaling pathway, negative regulation of immunoglobulin secretion, and programmed cell death (Supplementary File 9; Fig. 7). Nine genes that were downregulated in three of four CBD treatments (24 h) were enriched (FDR < 0.1) in BP associated with: cell chemotaxis and migration, regulation of amine transport, regulation of amino acid transport, regulation of monocyte chemotaxis and myeloid leukocyte migration. Single gene that was commonly affected by all CBD doses in 24-h treatment was *Mylk* that encode Myosin light chain kinase.

**Table 2:** Comparative analysis of biological processes and KEEG pathways overrepresented by different CBD treatments (concentrations) at 24-h treatment. Processes overrepresented by at least two treatments are presented along with the engaged genes

24-h treatment		
CBD concentration	Category	Genes
**Biological processes**		
CBD_0.325 CBD_1.5 CBD_3	Regulation of cell migration	ENSMUSG00000032531; ENSMUSG00000061353; ENSMUSG00000022836; ENSMUSG00000024190; ENSMUSG00000032714; ENSMUSG00000026822; ENSMUSG00000026981; ENSMUSG00000054836; ENSMUSG00000004951; ENSMUSG00000022836;
	Localization of cell	ENSMUSG00000032531; ENSMUSG00000061353; ENSMUSG00000040026; ENSMUSG00000022836; ENSMUSG00000024190; ENSMUSG00000026620; ENSMUSG00000032714; ENSMUSG00000061315; ENSMUSG00000026822; ENSMUSG00000026981; ENSMUSG00000054836; ENSMUSG00000022836;
	Cell motility	ENSMUSG00000032531; ENSMUSG00000061353; ENSMUSG00000040026; ENSMUSG00000022836; ENSMUSG00000024190; ENSMUSG00000026620; ENSMUSG00000032714; ENSMUSG00000061315; ENSMUSG00000026822; ENSMUSG00000026981; ENSMUSG00000054836
	Regulation of cell motility	ENSMUSG00000032531; ENSMUSG00000061353; ENSMUSG00000022836; ENSMUSG00000024190; ENSMUSG00000032714; ENSMUSG00000026822; ENSMUSG00000026981; ENSMUSG00000054836; ENSMUSG00000022836
	Regulation of cellular component movement	ENSMUSG00000032531; ENSMUSG00000061353; ENSMUSG00000022836; ENSMUSG00000024190; ENSMUSG00000032714; ENSMUSG00000038848; ENSMUSG00000026822; ENSMUSG00000026981 ENSMUSG00000054836; ENSMUSG00000022836
	Cell migration	ENSMUSG00000032531; ENSMUSG00000061353; ENSMUSG00000040026; ENSMUSG00000022836; ENSMUSG00000024190; ENSMUSG00000026620; ENSMUSG00000032714; ENSMUSG00000061315; ENSMUSG00000026822; ENSMUSG00000026981; ENSMUSG00000054836
	Regulation of locomotion	ENSMUSG00000032531; ENSMUSG00000061353; ENSMUSG00000022836; ENSMUSG00000024190; ENSMUSG00000032714; ENSMUSG00000038848; ENSMUSG00000026822; ENSMUSG00000026981; ENSMUSG00000054836; ENSMUSG00000022836;
CBD_0.325 CBD_1.5	Acute-phase response	ENSMUSG00000040026; ENSMUSG00000026981;
	Movement of cell or subcellular component	ENSMUSG00000032531; ENSMUSG00000061353; ENSMUSG00000040026; ENSMUSG00000022836; ENSMUSG00000024190; ENSMUSG00000026620; ENSMUSG00000032714; ENSMUSG00000038848; ENSMUSG00000061315; ENSMUSG00000026822; ENSMUSG00000026981; ENSMUSG00000032536; ENSMUSG00000054836
	Monocyte chemotaxis	ENSMUSG00000024190; ENSMUSG00000061353;
	Negative regulation of leukocyte migration	ENSMUSG00000024190; ENSMUSG00000061353;
	Locomotion	ENSMUSG00000032531; ENSMUSG00000061353; ENSMUSG00000040026; ENSMUSG00000022836; ENSMUSG00000024190; ENSMUSG00000026620; ENSMUSG00000032714; ENSMUSG00000038848; ENSMUSG00000061315; ENSMUSG00000026822; ENSMUSG00000026981; ENSMUSG00000054836;
	Negative regulation of amine transport	ENSMUSG00000038530; ENSMUSG00000026981;
	Negative regulation of anion transport	ENSMUSG00000038530; ENSMUSG00000026981;
	Regulation of amino acid transport	ENSMUSG00000038530; ENSMUSG00000026981;
	Regulation of amine transport	ENSMUSG00000038530; ENSMUSG00000061353; ENSMUSG00000026981
	Negative regulation of amino acid transport	ENSMUSG00000038530; ENSMUSG00000026981;
	Negative regulation of organic acid transport	ENSMUSG00000038530; ENSMUSG00000026981;
	Amine transport	ENSMUSG00000038530; ENSMUSG00000061353; ENSMUSG00000026981
	Regulation of monocyte chemotaxis	ENSMUSG00000024190; ENSMUSG00000061353;
	Gastrulation	ENSMUSG00000024190; ENSMUSG00000026981; ENSMUSG00000022330
CBD_1.5 CBD_3	Regulation of leukocyte differentiation	ENSMUSG00000027523; ENSMUSG00000052713; ENSMUSG00000048546; ENSMUSG00000004951;
**KEEG**		
CBD_0.325 CBD_1.5	Parkinson disease	ENSMUSG00000064363; ENSMUSG00000064367; ENSMUSG00000027523
	Thermogenesis	ENSMUSG00000064363; ENSMUSG00000064367; ENSMUSG00000027523
CBD_0.75 CBD_3	RNA degradation	ENSMUSG00000022283; ENSMUSG00000048546; ENSMUSG00000048546
CBD_1.5 CBD_3	Vascular smooth muscle contraction	ENSMUSG00000027523; ENSMUSG00000022836
	Gastric acid secretion	ENSMUSG00000027523; ENSMUSG00000022836;
	Platelet activation	ENSMUSG00000027523; ENSMUSG00000022836;
	Oxytocin signaling pathway	ENSMUSG00000027523; ENSMUSG00000022836;
	Salivary secretion	ENSMUSG00000027523; ENSMUSG00000027447
	Amoebiasis	ENSMUSG00000027523; ENSMUSG00000004951;
	Calcium signaling pathway	ENSMUSG00000027523; ENSMUSG00000022836;

Only six common genes have been affected by CBD at 6-h and 24-h treatments. They did not enrich any BP category but included genes associated with, e.g., cell chemotaxis, cell migration, acute-phase response, response to interleukin-1 (*Dusp1*, *Il1rn*, *Saa3*), and degradation of extracellular matrix proteins (*Mmp13*).

#### qPCR validation results

For the four validated genes, the Spearman rank correlation coefficient ranged from moderate to high, with values from 0.3 for *Gm8960* to 0.9 for *Ifitm2* (average of 0.625). However, none of these values was statistically significant, likely due to the low number of observations within the dataset. For all genes combined, the correlation coefficient was moderate (0.538) and statistically significant (*p* = 0.016) (Supplementary File 1). This suggests the reliability of the obtained results, especially when a laboratory-reproduced experiment was used as a validation control.

## Discussion

Cannabidiol (CBD) exhibits diverse biological effects in the brain, including the hypothalamus. Research on CBD’s impact on hypothalamic cells revealed its potential to modulate anxiety, stress responses, and other neurobiological functions. For example, in the case of anxiolytic effects, CBD microinjected into the ventromedial hypothalamus (VMH) in rats reduced panic attack-like behaviors and unconditioned fear-induced antinociception, potentially through CB1 receptor signaling (Khan et al. 2020). CBD was also found to enhance neurogenesis and exhibit neuroprotective effects. Repeated CBD administration in chronically stressed mice increased hippocampal neurogenesis and reduced anxiety-like behaviors (Campos et al. 2013). Additionally, CBD influenced histone modifications in the hypothalamus, which can affect gene expression. A recent study revealed that systemic administration of CBD modified the levels of certain histones (e.g., H3 K9ac) in the hypothalamus, indicating potential epigenetic effects of CBD (Pastrana-Trejo et al. 2021). An important action of CBD in the hypothalamus is the modulation of the stress-induced activation of the hypothalamic–pituitary–adrenal (HPA) axis (Viudez-Martínez et al. 2018). These complex responses to CBD in the hypothalamus require a molecular basis that can be observed only in isolated and simplified models, minimizing the complexity and multicellular interactions of brain structures.

In this study, we focused on the effects of CBD on proliferation, viability, and gene expression in a cellular model comprising hypothalamic neurons. We found that CBD stimulated cell viability, especially at longer incubation times (24 h) and at lower or intermediate CBD concentrations. Our results showed a noticeable decrease in apoptotic processes intensity with increasing CBD concentrations, although statistical significance was only observed at the highest CBD concentration. These effects were accompanied by moderate changes in cellular transcriptome profiles, with a greater number of genes affected by higher CBD concentrations and shorter incubation times (indicating a stronger initial response). The transcriptomic response varied between the two time points studied, with more genes upregulated at 6 h and a higher number of downregulated genes following prolonged CBD incubation.

All these observations require in depth analysis and comparison with currently available literature data.

### CBD affects hypothalamic neurons’ viability

The observation that CBD increases cell viability in in vitro cultured cells is atypical for healthy unchallenged cells, where CBD’s effect is predominantly associated with viability reduction (Pagano et al. 2020). The results obtained here contradict the findings from previously published data on hypothalamic and other neural cells (di Giacomo et al. 2020; Drummond-Main et al. 2023; Jantas et al. 2024). In most of these studies, CBD alone reduced cell viability primarily at higher concentrations, with no effect observed at lower CBD doses. However, this negative effect on cell viability was reversed when cells were challenged, e.g., with H2O2 to induce oxidative stress, where CBD treatment resulted in increased cell viability (di Giacomo et al. 2020; Jantas et al. 2024). These results indicate that CBD may exert effects that promote neuronal viability, as observed in this study on hypothalamic neurons. It remains unclear why this effect was observed here in unchallenged cells. It is possible that the CBD doses used are within a well-tolerated concertation range for mHypoA-2/12 mouse cell line, thus only the positive or neutral effect of CBD on their proliferation was observed. Alternatively, hypothalamic neural cells may react differently to CBD, similar to some other cell types such as PNT2 cells (Śledziński et al. 2023) or certain specific cancer cell lines (Omer et al. 2024), where CBD stimulates viability under basic culture conditions.

### CBD affects cell apoptosis

In this study, a trend toward reduction in apoptotic process intensity (as indicated by 3/7 caspase activity assay) was observed in mHypoA-2/12 cells. This findings, similarly to the viability trend, is unusual for healthy, untreated cells. Nevertheless, reducing apoptosis levels after CBD administration is not uncommon in cells exposed to various stressors. One study demonstrated that CBD protects dopaminergic neurons from oxidative stress and apoptosis by reducing reactive oxygen species (ROS) production and maintaining mitochondrial integrity, independently of CB1 and CB2 cannabinoid receptors (Mendivil-Perez et al. 2023). Furthermore, a review on CBD’s mechanisms in regulating apoptosis and autophagy suggests that these effects may vary based on biological context, cell type, and CBD concentration (Fu et al. 2023). Another study showed that CBD could mitigate perfluorooctane sulfonic acid (PFOS)-induced cardiomyocyte apoptosis by preserving mitochondrial dynamics and metabolic energy homeostasis (Wang et al. 2023). These documented protective and anti-apoptotic effects of CBD are well-supported in the literature, reinforcing the findings of this study.

### Transcriptomic evidence on apoptosis and viability regulation

The observed improvement in cell viability along with decreased apoptosis reflects neuroprotective potential and metabolic activity of CBD in hypothalamic neurons. To elucidate the underlying mechanisms at the transcriptome level, we conducted a detailed analysis of biological processes overrepresented by genes differentially expressed following various CBD treatments. Among the numerous processes affected by these treatments, those related to response to oxygen-containing compounds, cellular response to hypoxia, regulation of cell proliferation and cell cycle, regulation of programmed cell death, intrinsic apoptotic signaling pathway by p53 class mediator, DNA damage response, immune system processes, and regulation of metabolic processes were prominently altered in the 6-h treatment (Supplementary File 5; Supplementary File 7). In the 24-h treatment, processes involving regulation of cell migration, immune response, intrinsic apoptosis, and regulation of apoptosis were the most abundantly represented (Supplementary File 6; Supplementary File 9).

The analysis of these processes and the involved genes suggests that one of the important CBD actions in hypothalamic cells is the direct or indirect modulation of cellular apoptosis, particularly intrinsic apoptosis, potentially through the p53 pathway activity modulation (Supplementary File 5; Supplementary File 7). Intrinsic apoptosis involves mitochondrial proteins released in response to various stressors such as ultraviolet radiation, osmotic stress, growth factor withdrawal, chemotherapeutic agents, and natural compounds (Jan and Chaudhry 2019). Internal stimuli like high cytosolic Ca^2+^ concentrations, hypoxia, oxidative stress, and DNA damage also initiate mitochondrial pathway-mediated apoptosis (Carlsson et al. 2022). CBD’s activities in many of these intracellular processes have been previously documented (Ryan et al. 2009; Russo et al. 2019; Gross et al. 2021; Pereira et al. 2021). For instance, CBD has been shown to induce mitochondrial damage and cytochrome C release in cell lines derived from acute lymphoblastic leukemia of T lineage (T-ALL), and disrupt calcium homeostasis in those cells (Olivas-Aguirre et al. 2019). Additionally, CBD has been demonstrated to induce DNA damage in human-derived cell lines under conditions relevant to consumer exposure (Russo et al. 2019). While examining the key genes that were altered in our dataset and had major contributions to the regulation of apoptotic pathways, we found that the most important hub genes in 6-h treatment were *Bbc3* (PUMA; which was downregulated by two lower CBD doses), and *Mdm2* (that was upregulated by lowest and intermediate CBD doses). The Bbc3 (Bcl-2-binding component 3), also known as the p53 upregulated modulator of apoptosis (PUMA) is a pro-apoptotic protein, a member of the Bcl-2 protein family (Nakano and Vousden 2001). Upon activation by p53, PUMA interacts with antiapoptotic Bcl-2 family members, thereby releasing Bax and/or Bak to signal apoptosis to the mitochondria (Han et al. 2001). Subsequent mitochondrial dysfunction triggers the caspase cascade, culminating in cell death. In contrast, mouse double minute 2 homolog (Mdm2), also known as E3 ubiquitin-protein ligase, is a critical negative regulator of the p53 tumor suppressor (Mendoza et al. 2014). Mdm2 functions as both an E3 ubiquitin ligase, recognizing the N-terminal trans-activation domain (TAD) of p53, and an inhibitor of p53 transcriptional activation, thereby suppressing p53-mediated apoptosis (de Rozieres et al. 2000). Mdm2 has also been shown to promote proliferation and inhibit apoptosis in pituitary adenoma cells by directly interacting with p53 (Wang et al. 2020). Therefore, the interplay between Bbc3 (PUMA) and Mdm2 seems to be pivotal for CBD-mediated regulation of apoptosis via the p53 pathway. In the anticipated mechanism modulated by CBD, *PUMA* expression is inhibited directly or indirectly by CBD, thereby suppressing apoptosis, while the upregulation of *Mdm2* aims to negatively regulate p53, further mitigating excessive apoptotic activity. These changes in gene expression align with the observed effects of CBD on viability and apoptosis in hypothalamus cells in this study.

The other genes identified in this study as differentially expressed, which may significantly impact apoptosis processes, proliferation regulation, and immune responses, include among others *Cdkn1a* (p21, Cyclin-dependent kinase inhibitor 1 A), *Ndrg1* (N-myc downstream–regulated gene), and *Smad3*, which contributes to the maintenance of genomic integrity, cell cycle regulation, and apoptosis. The regulation of *Cdkn1a* and *Ndrg1* by p53, and their roles in cellular stress and apoptosis, underscore their critical functions in preserving cellular integrity (Kreis et al. 2019; Schonkeren et al. 2019). *Cdkn1a* is a direct transcriptional target of p53. Upon cellular stress signals such as DNA damage, p53 binds to the promoter region of *Cdkn1a*, thereby inducing its expression. This leads to the inhibition of cyclin-dependent kinases (CDKs), resulting in cell cycle arrest at the G1 phase. Such cell cycle arrest allows for DNA damage repair before cell division proceeds, thereby preventing the propagation of mutations (Kreis et al. 2019). Smad3, a member of the SMAD family of proteins involved in TGF-β signaling, interacts with both p53 and Cdkn1a. In some contexts, Smad3 can directly regulate p21 expression, contributing to cell cycle regulation and apoptosis. Additionally, Smad3 signaling can intersect with p53 pathways to modulate cellular responses to stress and DNA damage. Smad3 involvement in TGF-β signaling adds another layer of complexity to the regulation of cell cycle, apoptosis, and stress response. Its interaction with both p53 and Cdkn1a underscores the intricate crosstalk between different signaling pathways in coordinating cellular responses to various stimuli (Wang et al. 2016a).

CBD’s ability to downregulate the expression of genes *Cdkn1a, Ndrg1, Smad3, and Bbc2* can have both beneficial and potentially adverse effects. It may offer advantages such as anti-inflammatory and antitumor properties, as well as neuroprotective effects. However, there are risks, including potential disruption of cellular homeostasis, impaired cellular defense mechanisms, and unintended effects on other cellular processes. Further research would be needed to fully understand the consequences of modulating all mentioned genes and to evaluate the therapeutic potential of such interventions, particularly in the context of neurodegenerative diseases.

At 24 h of treatment, the set of genes associated with apoptosis significantly differed, suggesting the alteration of downstream elements of the intrinsic apoptosis mechanism with prolonged CBD treatments. Among the apoptosis-related genes, that were altered predominantly at 24-h treatment, there were for example *Hspb1* (Heat shock protein beta-1), which product is known to inhibit apoptosis by stabilizing the actin cytoskeleton and preventing cytochrome c release from mitochondria (Park et al. 2002); *Tmbim6* (Transmembrane BAX inhibitor motif containing 6), encoding an anti-apoptotic protein that inhibits mitochondrial calcium uptake and oxidative stress-induced cell death (Kim et al. 2021); *Cyld* (Cylindromatosis), a tumor suppressor gene that negatively regulates NF-κB signaling, promoting apoptosis by removing K63-linked polyubiquitin chains from target proteins (Fernández-Majada et al. 2016); and *Dusp1* (Dual specificity phosphatase 1 also known as MAP kinase phosphatase-1), involved in the negative regulation of MAPK signaling, which can lead to the inhibition of apoptosis (Wang et al. 2016b). These and other apoptosis-related genes altered by CBD in this study suggest that CBD’s effect on intrinsic apoptosis persists over time, though later stages may involve different molecular mechanisms compared to short-term treatments.

The mechanism by which CBD affects cell viability, particularly at the transcriptome level, remains unclear. However, our findings suggest that CBD can stimulate the expression of genes associated with mitochondrial respiratory chain complex I, providing new insights into this process. In our study, the two most significantly affected genes were *mt-Nd4* and *mt-Nd5*. These genes encode NADH-ubiquinone oxidoreductase chain proteins, which are subunits of NADH dehydrogenase (ubiquinone). This enzyme, located in the inner mitochondrial membrane, is the largest of the five complexes in the electron transport chain. NADH dehydrogenase and NAD (nicotinamide adenine dinucleotide) play a crucial role in regulating cell viability through its involvement in energy metabolism and protection against oxidative stress (Čermáková et al. 2021). Our findings align with previous studies on the brains of CBD-treated rats, where increased mitochondrial calcium accumulation enhanced the activity of calcium-sensitive dehydrogenases. This, in turn, promoted NADH availability and enhanced oxidative phosphorylation (Valvassori et al. 2013). Our results further support the broader impact of CBD on mitochondrial metabolism across various cell types (Olivas-Aguirre et al. 2019).

### Modulation of stress-like neurons’ responses by CBD

CBD induces cellular responses with transcriptomic signatures resembling known stress responses, primarily evident at the 6-h treatment (initial response). Among the cellular processes overrepresented by DEGs, we identified ones associated with general stress response, cellular response to external stimuli, cellular response to chemical stimuli, response to inorganic substances, interleukin-27-mediated signaling pathway, response to hypoxia, DNA damage response signal transduction mediated by p53 resulting in cell cycle arrest, response to endoplasmic reticulum stress, response to oxidative stress, and others. While the cellular reactions to external and chemical stimuli are obvious, other processes require further analysis and explanation.

An intriguing CBD-associated cellular response is the interleukin-27-mediated signaling pathway, enriched with genes altered by the lowest CBD concentration. Interleukin-27 (IL-27) is recognized as a pivotal regulator of T cell activation and differentiation, influencing T cell responses in autoimmune conditions within the central nervous system (Iwasaki et al. 2015). Recent evidence also suggests its neuroprotective properties in the retina and brain (Nortey et al. 2022). IL-27, secreted by and interacting with infiltrating microglia, macrophages, astrocytes, and neurons, enhances neuronal survival by modulating pro- and anti-inflammatory cytokines, neuroinflammatory pathways, oxidative stress, apoptosis, autophagy, and epigenetic changes (Nortey et al. 2022). The pathway-associated genes such as *Oasl1* and *Oasl2* were upregulated by lower CBD doses in this research. Oasl proteins (2′−5′-oligoadenylate synthase) may indirectly affect neurodegeneration by regulating inflammatory processes and immune responses in the brain (Ghosh et al. 2019). However, their specific roles and regulatory mechanisms in the context of CBD action, and their implications for CBD consumption, warrant further investigation.

### CBD’s effect on extracellular matrix

The genes altered by CBD, primarily in short-term treatments, enrich cellular components such as the extracellular region, extracellular matrix (ECM), and collagen-containing extracellular matrix. The interaction between CBD and the ECM has been previously documented, mainly in different types of fibroblastic cells. It has been shown that CBD, at lower concentrations, increases the production of metalloproteinases (MMPs), while the highest concentrations decrease both the production of MMPs and MMP-2 protein activity (Rawal et al. 2012). A previous transcriptome analysis revealed that CBD pre-treatment enriched genes and functional associations between proteins mainly related to ECM organization and cell interactions in the mouse brain (Prieto et al. 2023). In our previous transcriptomic study on human dermal fibroblasts, we found that CBD affected several genes connected with ECM formation (especially its collagen constituent), which can have serious implications for the fibrosis process (in press). In this study, several genes connected to ECM functioning were altered, the most significant being most likely *Mmp3* (downregulated by all 6 h CBD treatments), *Mmp13* (downregulated by all 24-h treatments and one 6-h treatment), *Timp1* (downregulated by all 6-h treatments), and *Col11a1* (downregulated by a single 6-h treatment).

Mmp3 (matrix metalloproteinase-3) is a matrix metalloproteinase, a proteolytic enzyme capable of degrading many ECM components, including proteoglycans, collagens (types III, IV, V, IX, and X), laminin, and fibronectin. Mmp3 plays a key role in tissue remodeling, wound healing, and pathological processes such as inflammation and cancer development (Kandhwal et al. 2022). Its activity is tightly regulated because excessive ECM degradation can lead to diseases such as arthritis and cancer (Mehner et al. 2015). Research suggests that CBD can modulate the activity of MMPs (Gęgotek et al. 2021), which leads to reduced inflammation and protection of the ECM from excessive degradation. Similarly, Mmp13 (matrix metalloproteinase-13) degrades type II collagen as well as other collagen types. It is particularly important in the remodeling of cartilage and bones (Hu and Ecker 2021). One study suggests that CBD derivate can reduce the expression *Mmp13* (Jin et al. 2023) and now we further confirm this in this study.

Timp1 (tissue inhibitor of metalloproteinases-1) inhibits the activity of metalloproteinases such as Mmp3 and Mmp13, protecting the ECM from excessive degradation (Brew et al. 2000). Previous research has shown that CBD can increase the expression of Timp1, supporting the protection of the ECM against degradation and potentially benefiting the treatment of inflammation and cancer (Solinas et al. 2012, 2013).

Finally, Col11a1 (collagen type XI alpha 1 chain) is a component of type XI collagen, which supports the structural integrity of cartilage (Shi et al. 2022). In this study, we found that its transcript abundance can be modulated by CBD.

In summary, all mentioned ECM-related genes participate in the process of connective tissue remodeling, where their coordinated action enables the dynamic maintenance and reconstruction of the ECM. Mmp3 and Mmp13 are responsible for the degradation of ECM components, while Timp1 regulates their activity, protecting the ECM against excessive degradation. Col11a1, on the other hand, is important for maintaining the structure of the ECM. This complex interaction is crucial for tissue health and regeneration, and the modulation of this process by CBD can have various implications, particularly related to pathologies such as degenerative joint diseases and cancer.

### Transcriptomic insights into the regulation of hypothalamic functions by CBD

In the analysis of genes altered by CBD after 24 h of treatment, we identified those enriched in the dopamine and serotonin biosynthetic processes, as well as in the dopamine receptor signaling pathway. Dopamine is crucial in the hypothalamus, influencing neuroendocrine and autonomic functions. It regulates the HPA axis, with D1 and D2 receptors activating the HPA axis in response to severe stress in rats (Belda and Armario 2009). Given these roles, we focused on genes involved in these pathways and found that *Aldh2*, protein of which assists in dopamine and serotonin synthesis, was upregulated by the lowest CBD dose after 24 h of treatment. What is more, a *Rgs4* gene engaged in the regulation of the dopamine receptor signaling pathway was downregulated in most of the 24 h CBD treatments. Aldh2 (aldehyde dehydrogenase 2 family member) belongs to the aldehyde dehydrogenase family of proteins and is essential in the major oxidative pathway of alcohol metabolism (Adams and Rans 2013; Chen et al. 2014). This links CBD’s effect on neural cells to its presumed positive properties in therapies for alcohol use disorders (De Ternay et al. 2019), though further studies are needed to explore this aspect. Regarding the *Rgs4* gene (regulator of G protein signaling 4), its downregulation observed in most 24-h CBD treatments may affect dopamine D2 and D3 receptors, which are major targets for current antipsychotic drugs (Min et al. 2012). Studies using cDNA microarrays have consistently shown decreased *RGS4* expression in the prefrontal cortex of subjects with schizophrenia, suggesting implications for CBD in anxiety and mental disorder therapies (McGuire et al. 2018).

## Conclusions

The study evaluated the effects of CBD on adult-derived hypothalamic neurons, focusing on cell proliferation, survival, and gene expression. Our results demonstrate that CBD significantly enhances neuronal viability, particularly with extended incubation times of 24 h and at lower to intermediate concentrations. CBD showed a trend toward reducing apoptosis, with statistical significance achieved at the highest concentration. CBD also caused moderate changes in the cellular transcriptome, with more genes affected by higher concentrations and shorter incubation times, indicating a stronger initial response. The gene expression profile varied between time points, with an increase in upregulated genes observed at 6 h and a predominance of downregulated genes with prolonged incubation. Detailed analysis of gene-associated processes showed that CBD altered several cellular responses, resulting in intrinsic apoptosis suppression, immune response modulation, extracellular matrix reorganization, and other cellular mechanism alterations. We found that CBD primarily affects intrinsic apoptosis through p53 modulation, likely by influencing the expression of *Bbc3*, *Mdm2*, *Cdkn1a*, and *Smad3* genes. CBD also impacted several genes related to ECM organization, including important MMPs (*Mmp-3*, *Mmp-13*) and their inhibitors (such as *Timp1*), as well as collagen components. Additionally, CBD-influenced genes associated with serotonin and dopamine biosynthesis, including the *Aldh2* gene, link CBD’s action in hypothalamic neurons to its proposed benefits in treating alcohol use disorders. These findings confirm the hypothalamus as an important target for CBD actions, highlighting its diverse impact on the central nervous system. Future research will investigate the initiation and underlying mechanisms of these action in more detail.

## Supplementary Information

Below is the link to the electronic supplementary material.

Supplementary file1 Primers used for qPCR RNA-Seq validation and obtained correlation coefficients (XLSX 10 KB)

Supplementary file2 Sequencing reads and mapping statistics (XLSX 15 KB)

Supplementary file3 Differential expression analysis for cells treated with different concentrations of CBD for 6 and 24 h (including genes regulation tables) (XLSX 17489 KB)

Supplementary file4 Comparative analysis for genes affected by different CBD concentrations and differ treatment times (including altered genes annotations) (XLSX 24 KB)

Supplementary file5 Altered genes (by all CBD concentrations separately at 6 h treatment) overrepresentation tests in GO biological processes and KEEG pathways categories (including comparative reanalysis of processes/pathways) (XLSX 444 KB)

Supplementary file6 Altered genes (by all CBD concentrations separately at 6 h and 24 h treatments) overrepresentation tests in GO cellular components categories (XLSX 22 KB)

Supplementary file7 Altered genes (by all CBD concentrations jointly at 6 h treatment) overrepresentation tests in GO biological processes (HTML 493 KB)

Supplementary file8 Altered genes (by all CBD concentrations separately at 24 h treatment) overrepresentation tests in GO biological processes and KEEG pathways categories (including comparative reanalysis of processes/pathways) (XLSX 438 KB)

Supplementary file9 Altered genes (by all CBD concentrations jointly at 24 h treatment) overrepresentation tests in GO biological processes (HTML 323 KB)

## Data Availability

Raw sequencing reads and read counts were deposited in the Gene Expression Omnibus (GEO) and Sequence Read Archive (SRA) databases of the National Center for Biotechnology Information (NCBI) under accession number GSE270378.
